# Weakly haemolytic variants of *Brachyspira hyodysenteriae* newly emerged in Europe belong to a distinct subclade with unique genetic properties

**DOI:** 10.1186/s13567-019-0639-x

**Published:** 2019-03-07

**Authors:** Roderick M. Card, Tom La, Eric R. Burrough, Richard J. Ellis, Javier Nunez-Garcia, Jill R. Thomson, Maxime Mahu, Nyree D. Phillips, David J. Hampson, Judith Rohde, Alexander W. Tucker

**Affiliations:** 1 Department of Bacteriology, Animal and Plant Health Agency, Addlestone, UK; 2 School of Veterinary and Life Sciences, Murdoch University, Perth, Australia; 3 Veterinary Diagnostic Laboratory, Iowa State University, Ames, USA; 4 Surveillance and Laboratory Services Department, Animal and Plant Health Agency, Addlestone, UK; 5 Veterinary Services, Scotland’s Rural College, Penicuik, UK; 6 Department of Pathology, Bacteriology and Avian Diseases, Faculty of Veterinary Medicine, Ghent University, Ghent, Belgium; 7 Department of Infectious Diseases and Public Health, City University of Hong Kong, Kowloon Tong, Hong Kong; 8 Institute for Microbiology, University of Veterinary Medicine, Hannover, Germany; 9 Department of Veterinary Medicine, University of Cambridge, Cambridge, UK; 10 Present Address: Genomics Medicine Ireland, Dublin, Ireland

## Abstract

*Brachyspira* (*B.*) *hyodysenteriae* is widespread globally, and can cause mucohaemorrhagic colitis (swine dysentery, SD) with severe economic impact in infected herds. Typical strains of *B. hyodysenteriae* are strongly haemolytic on blood agar, and the haemolytic activity is believed to contribute to virulence in vivo. However, recently there have been reports of atypical weakly haemolytic isolates of *B. hyodysenteriae* (whBh). In this study, 34 European whBh and 82 strongly haemolytic isolates were subjected to comparative genomic analysis. A phylogenetic tree constructed using core single nucleotide polymorphisms showed that the whBh formed a distinct sub-clade. All eight genes previously associated with haemolysis in *B. hyodysenteriae* were present in the whBh. No consistent patterns of amino acid substitutions for all whBh were found in these genes. In contrast, a genome region containing six coding sequences (CDSs) had consistent nucleotide sequence differences between strongly and whBh isolates. Two CDSs were predicted to encode ABC transporter proteins, and a TolC family protein, which may have a role in the export of haemolysins from *B. hyodysenteriae.* Another difference in this region was the presence of three CDSs in whBh that are pseudogenes in strongly haemolytic isolates. One of the intact CDSs from whBh encoded a predicted PadR-like transcriptional repressor that may play a role in repression of haemolysis functions. In summary, a sub-clade of whBh isolates has emerged in Europe, and several genomic differences, that potentially explain the weakly haemolytic phenotype, were identified. These markers may provide targets for discriminatory molecular tests needed in SD surveillance.

**Electronic supplementary material:**

The online version of this article (10.1186/s13567-019-0639-x) contains supplementary material, which is available to authorized users.

## Introduction

Swine dysentery (SD) is a bacterial disease of pigs characterised by development of a severe mucohaemorrhagic colitis that mainly occurs in animals in the growing and finishing stages. The disease is widely distributed globally and causes substantial production losses in infected swine herds. Consequently, accurate surveillance for SD-causing bacteria is an important element of disease prevention and trade control in many countries. Three species of anaerobic intestinal spirochaetes have been shown to be capable of causing SD, of which *Brachyspira hyodysenteriae* is the most common and widespread [1]. *B. suanatina* and *B. hampsonii*, the other two species that can cause SD, share with *B. hyodysenteriae* the capacity to cause strong beta-haemolysis on blood agar plates [2, 3]. Other *Brachyspira* species that colonise pigs but which do not induce SD are all weakly haemolytic; these include the pathogenic *B. pilosicoli*, non-pathogenic *B. innocens*, and *B. murdochii* and *B. intermedia* which may have some mild pathogenic potential [4]. The difference in extent of haemolysis between those species that do or do not induce the mucohaemorrhagic colitis of SD helps to reinforce the likelihood that the pathogenesis of SD involves the activities of one or more molecules associated with or correlated with the pathways that lead to the production of strong beta-haemolysis [1].

Studies have shown that a protein with haemolytic activity extracted from the supernatant of *B. hyodysenteriae* cultures can cause lesions resembling those seen in SD when placed in ligated ileal and colonic loops in germ-free pigs [5], and in the caecum in a murine model of SD [6]. These observations suggest that this haemolysin is involved in lesion formation, although it does not exclude the possibility that other mechanisms also are involved in aspects of the pathogenesis of SD. For example, Witters and Duhamel demonstrated that *B. hyodysenteriae* strain B78^T^, which is less virulent than strains B204 and B234, had higher haemolysin titres than the latter two strains [7]. An effect of population density on the strength of haemolysis was also shown in that study, with some isolates demonstrating stronger in vitro haemolysis at higher cell densities [7]. In other studies the extracted *B. hyodysenteriae* haemolysin has been described as having a wide variety of molecular masses depending on the purification methods used in the study [8]; consequently, some uncertainty remains about the precise identity of the haemolysin(s).

Eight genes have been annotated as potentially being involved in the pathway leading to expression of the strong beta-haemolytic phenotype of *B. hyodysenteriae*. These include *hlyA/*ACP, encoding an acyl carrier protein which exhibits β-haemolytic activity [8]; *tlyA*, encoding a protein having homology with pore-forming haemolysins [9]; *tlyB*, encoding Clp protease [10, 11]; *tlyC*, encoding haemolysin C [10, 11]; *haemolysin III*, encoding a predicted channel forming protein; *haemolysin activation protein* gene, encoding a protein with similarity with haemolysin C; *haemolysin channel protein* gene, encoding an integral membrane protein; and a *haemolysin* gene [12, 13]. Of these, *hlyA*, *tlyA*, *tlyB* and *tlyC* have all been shown to confer a beta-haemolytic phenotype when transferred to non-haemolytic *Escherichia coli* strains [8, 11]. Some or all of these genes have also been detected in strains of the weakly haemolytic *Brachyspira* species, which complicates interpretation of their presence and role in production of strong beta-haemolysis [14, 15]. Indeed, it remains unclear whether the weakly haemolytic species produce different haemolysin(s) to those of *B. hyodysenteriae*, or whether weak haemolysis is caused by the same molecule as produced by *B. hyodysenteriae* but whose expression, processing and/or export is comparatively reduced.

In recent years a number of “atypical” weakly haemolytic isolates of *B. hyodysenteriae* (whBh) have been recovered from pigs in European and Australian herds in the absence of SD and where there is little or no diarrhoeal disease [16–18]. One of these whBh failed to cause disease, when used to experimentally infect pigs [19], and this finding increased the available evidence suggesting that the expression of strong beta-haemolysis has an important role in the pathogenesis of SD. When investigating the reason for the weakly haemolytic phenotype, a few single nucleotide polymorphisms (SNPs) were inconsistently found in some of the annotated haemolysin genes [18]. In another study, a whBh isolate had 10 amino acid substitutions in the haemolysin III protein and five in the haemolysin activation protein compared to strongly beta-haemolytic reference strain WA1, and it had a five nucleotide insertion disrupting the promoter site of the *hlyA* gene [17].

Given the current uncertainty about which genes and pathways are involved in creating the strongly beta-haemolytic phenotype in *B. hyodysenteriae*, this study took a comparative genomics approach to analyse whole genomic sequences (WGSs) of strongly and weakly beta-haemolytic *B. hyodysenteriae* recovered from pigs in Europe. A total of 116 WGSs were examined, comprising 77 published genomes (of which four were from whBh isolates) and 39 newly sequenced genomes (including 31 whBh isolates). The aim of this work was to determine the genetic relationships between the available European whBh strains and other more typical strains, and to identify consistent differences in gene content and/or predicted gene function between strongly and weakly beta-haemolytic isolates that could help to elucidate the pathways involved in production of strong beta-haemolysis in *B. hyodysenteriae.*

## Materials and methods

### Bacterial isolates

Thirty-nine isolates of *B. hyodysenteriae* were sequenced for this study, including the previously described whBh isolate D28 [18]. Eight isolates had a strongly haemolytic phenotype in culture of which four were from pigs with SD, three from pigs with diarrhoea and one from a pig with an unknown clinical background. The remaining 31 isolates had a weakly haemolytic phenotype on blood agar. Twenty-four isolates were from samples collected for surveillance in herds, which presented no clinical signs of SD, six were from pigs with diarrhoea and one lacked anamnestic information. Isolates were collected from diagnostic samples (*n* = 15) or samples collected to assess infection status as part of disease control (*n* = 24). Samples were of three types: excreted faecal samples not collected directly from live pigs (*n* = 2); rectal swabs collected from individual live pigs by veterinary surgeons, which did not require anaesthesia, and was not harmful to the pigs (*n* = 30); intestinal contents collected from dead pigs (*n* = 7; no animals were euthanised specifically for this study). This sampling strategy is part of the normal veterinary diagnostic investigation of this type of disease on a farm and as such is not for scientific purposes. All sampling was undertaken strictly according to the applicable national regulations in each country. Details of the newly sequenced isolates and the four previously sequenced whBh isolates are summarised in Table 1.

**Table 1:** **Summary of the 39** ***B. hyodysenteriae*** **isolates sequenced in this project**

Isolate	Year	Country	Farm	Haemolysis strength	Clinical background	Sequence type	Plasmid type	Clone number	References
JR88	2016	Germany	Farm V	Weak	Unknown	246	1	1	This study
BH23	2010	UK	Farm BF (UK)	Weak	Surveillance	167	1	2	[32]
D28	2011	Belgium	Farm B1 (Belgium)	Weak	Diarrhoea	172	1	3	[18]
JR11	2014	Germany	Farm C	Weak	Surveillance	134	1	4	[17]
JR12	2014	Germany	Farm C	Weak	Surveillance	134	1	4	[17]
JR13	2014	Germany	Farm C	Weak	Surveillance	134	1	4	[17]
JR15	2014	Germany	Farm M	Weak	Diarrhoea	135	1	5	This study
JR47	2015	Germany	Farm R1	Weak	Surveillance	191	1	6	This study
JR48	2015	Germany	Farm R1	Weak	Surveillance	191	1	6	This study
JR51	2015	Germany	Farm R1	Weak	Surveillance	191	1	6	This study
JR53	2015	Germany	Farm R1	Weak	Surveillance	191	1	6	This study
JR54	2015	Germany	Farm R1	Weak	Surveillance	191	1	6	This study
JR56	2015	Germany	Farm R1	Weak	Surveillance	191	1	6	This study
JR57	2015	Germany	Farm R1	Weak	Surveillance	191	1	6	This study
JR58	2015	Germany	Farm R1	Weak	Surveillance	191	1	6	This study
JR59	2015	Germany	Farm R1	Weak	Surveillance	191	1	6	This study
JR60	2015	Germany	Farm R2	Weak	Surveillance	191	1	6	This study
JR61	2015	Germany	Farm R2	Weak	Surveillance	191	1	6	This study
JR63	2015	Germany	Farm R2	Weak	Surveillance	191	1	6	This study
JR64	2015	Germany	Farm R3	Weak	Surveillance	191	1	6	This study
JR65	2015	Germany	Farm R4	Weak	Diarrhoea	191	1	6	This study
JR76	2016	Germany	Farm R4	Weak	Diarrhoea	191	1	6	This study
JR49	2015	Germany	Farm R1	Weak	Surveillance	192	1	7	This study
JR50	2015	Germany	Farm R1	Weak	Surveillance	192	1	7	This study
JR71	2016	Germany	Farm W2	Weak	Surveillance	219	1	8	This study
AWT1-5	2016	Germany	Farm W1	Weak	Surveillance	219	1	8	This study
AWT1-3	2016	Germany	Farm W2	Weak	Surveillance	219	1	8	This study
AWT1-6	2016	Germany	Farm W3	Weak	Surveillance	219	1	8	This study
AWT1-7	2016	Germany	Farm W3	Weak	Surveillance	219	1	8	This study
JR79	2015	Germany	Farm W4	Weak	Diarrhoea	219	1	8	This study
JR80	2015	Germany	Farm W4	Weak	Diarrhoea	219	1	8	This study
JR73	2017	Germany	Farm Z	Weak	Surveillance	Partial^1^	4	9	This study
JR74	2017	Germany	Farm Z	Weak	Surveillance	Partial^1^	4	9	This study
JR77	2016	Germany	Farm I	Weak	Surveillance	Partial^2^	4	10	This study
JR72	2017	Germany	Farm II	Weak/strong^a^	Surveillance	Partial^3^	1	n/a	This study
AWT2–4	2015	Spain	Farm X	Strong	Swine dysentery	Partial^4^	4	n/a	This study
JR22	2014	Germany	Farm N	Strong	Diarrhoea	52	1	n/a	This study
JR31	2014	Germany	Farm O	Strong	Unknown	112	4	n/a	This study
JR32	2014	Germany	Farm P	Strong	Diarrhoea	52	1	n/a	This study
JR42	2014	Germany	Farm Q	Strong	Diarrhoea	139	4	n/a	This study
JR96	2015	Germany	Farm S	Strong	Swine dysentery	52	1	n/a	This study
JR97	2015	Germany	Farm T	Strong	Swine dysentery	52	1	n/a	This study
JR98	2015	Germany	Farm U	Strong	Swine dysentery	112	4	n/a	This study

Details of the four previously sequenced weakly haemolytic *B. hyodysenteriae* are included for comparison. Farm of origin is given as an anonymised letter code, together with year of sampling, country of origin and haemolysis phenotype on TSA plates. Sequence type (ST), clone number, and plasmid type (PT) are indicated.

^a^Isolate JR72 was weakly haemolytic on TSA plates, but strongly haemolytic in an in vitro haemolysis assay at high optical density (OD_600_ = 0.550 ± 0.05).

Partial^1^ (*adh* not detected; *alp*: not detected; *est3*; *gdh21*; *glpK40*; *pgm2*; *thi21*).

Partial^2^ (*adh* not detected; *alp* not detected; *est29*; *gdh1*; *glpK10*; *pgm*~*22*; *thi38*).

Partial^3^ (*adh2*; *alp* not detected; *est*~*11*; *gdh7*; *glpK8*; *pgm3*; *thi17*).

Partial^4^ (*adh2*; *alp* not detected; *est5*; *gdh19*; *glpK*~*19*; *pgm3*; *thi3*).

Isolates from Germany were recovered from primary cultures on selective Trypticase Soy Agar (TSA) supplemented with 0.1% yeast extract, 6 μg/mL vancomycin, 6.25 μg/mL colistin, 12.5 μg/mL rifampicin, 15.25 μg/mL spiramycin, 200 μg/mL spectinomycin, and 5% bovine blood [20] and on Columbia blood agar (CBA), all supplied by Oxoid, Wesel, Germany, and were incubated anaerobically in an AnaeroJar with an AnaeroGen gas generator (Oxoid) at 42 °C for 6 days. They were initially assigned to a *Brachyspira* species by *nox*-RFLP [21], species-specific PCR [22] and partial *nox*-gene-sequencing using the same primers as for *nox*-RFLP. Six isolates were also identified using biochemical identification according to the strength of haemolysis and the determination of α-galactosidase, α-glucosidase, β-glucosidase, and hippurate activity using established procedures [23]. Species identification by MALDI-ToF mass spectrometry was performed as described previously [24]. A custom library encompassing the main spectra of the commercial Bruker library, the main spectra validated by Warneke et al. [24] and spectra from *B. pilosicoli* P43/6/78, *B. alvinipulli* AN1268/3/04, *B. murdochii* C301, *B. innocens* C336, *B. hampsonii* genomic group III 3824-15x/14, *B. hamsponii* genomic group I 5369-1x/12, *B. intermedia* AN26/93, *B. suanatina* AN4859/03, *B. hyodysenteriae* B204 and *B. hyodysenteriae* B78 was used for identification of isolates. Strength of haemolysis of pure cultures and ring phenomenon [25] were preliminarily assessed on TSA with 10% bovine blood at 42 °C with isolates not showing a delineated zone of translucency around an agar punch hole being designated as weakly haemolytic. In our hands, growth on TSA with 10% bovine blood produces a more pronounced ring phenomenon than growth on 5% ovine blood as described originally [25].

### In vitro assay for strength of haemolysis

The strength of haemolysis for single whBh isolates belonging to all clones except clones 2 and 3 (see “Results” for definition) was examined using an in vitro haemolysis assay, essentially as described previously [18], but with culture optical density normalised to OD_600_ 0.250 (± 0.05) and sheep erythrocytes used as the test cells, because they are commonly used in haemolysis assays and therefore provide a comparable system across studies. Triton-X 2% (complete haemolysis) and the strongly haemolytic B78^T^ *B. hyodysenteriae* type strain served as positive controls, and the strongly haemolytic field isolate JR96 also was tested. *B. innocens* strain C336 (kindly provided by Fellström [26, 27]) and fresh brain heart infusion broth with 10% foetal calf serum served as negative controls (weak haemolysis and no haemolysis, respectively). Subsequently, B78^T^, JR11 and JR72 were tested again in the same system but with the culture optical density normalised to OD_600_ 0.550 (± 0.05). One-way ANOVA with Bonferroni correction was used to test for significance of difference in strength of haemolysis between the positive control B78^T^ and test isolates, with *p* < 0.05 considered significant.

### Whole genome sequencing and analysis

To obtain material for sequencing, isolates were cultured on Fastidious Anaerobe Agar or Tryptic Soy Agar (TSA) incorporating either 10% bovine blood or 5% sheep blood and 1% yeast extract, and incubated under anaerobic conditions at 37–38 °C for 3 to 5 days. DNA extracts were prepared from cell pellets using Prepman Ultra (Life Technologies, UK) or the DNeasy Blood and Tissue Kit (Qiagen, Hilden, Germany), according to the manufacturers’ protocol. Nextera XT libraries were prepared for WGS (Illumina, Lesser Chesterford, UK) and sequenced on an Illumina MiSeq platform using either v2 chemistry and a 2 × 150 bp paired-end protocol or using v3 chemistry and 300 bp paired-end protocol. The raw sequences for each isolate were analysed with the Nullarbor pipeline (version 1.20; [28]) using the closed genome of WA1 [12] as reference, and SPAdes version 3.9.0 [29] and Prokka version 1.11 [30] for genome assembly and annotation respectively. SNPs with respect to WA1 were calculated using Snippy (version 3.0) within the Nullarbor pipeline. SNPs were filtered using minimum reads equal to 10, the minimum proportion of those reads, which must differ from WA1 equal to 0.9, and minimum variant call quality equal to 100. A maximum likelihood phylogenetic tree using the core genome SNPs was constructed within the Nullarbor pipeline using FastTree (version 2.1.8; [31]). The reliability of each split in the tree was calculated by FastTree using the Shimodaira-Hasegawa test with 1000 resamples [31]. The raw data from the published genomes of 77 *B. hyodysenteriae* isolates from swine [13, 17, 32, 33] were included in this analysis; of these isolates four had been reported as weakly haemolytic: JR11–JR13 [17] and BH23 [32]. Species were assigned by Kraken [34] (version 0.10.5-beta) and gene presence/absence lists were generated using Roary [35]. Scoary [36] was used to perform a genome-wide association study (GWAS) to identify genes and core genome SNPs having significant association with weak haemolysis (*p* < 0.05; after Bonferroni correction for multiple tests). Coding sequences (CDSs) identified in this manner were extracted from the de novo assemblies using custom scripts, examined by blastn [37] and aligned using ClustalV in MegAlign (DNASTAR, Madison, WI, USA).

Additionally the genome sequences from the 77 published and the 39 newly sequenced isolates were analysed with SeqFinder [38] using the genome of the WA1 chromosome (Accession number NC_012225) and plasmid (Accession number NC_012226) as references, as described previously [32]. Isolate sequence type (ST) was determined by extracting the seven house-keeping genes of the *B. hyodysenteriae* MLST scheme (*adh*, *alp*, *est*, *gdh*, *glpK*, *pgm*, and *thi*) [39] and interrogation of the PubMLST database [40]. The sequences of the eight recognised haemolysis associated genes [17] were extracted, aligned using ClustalV in MegAlign (DNASTAR) and examined for amino acid changes uniquely present in weakly haemolytic isolates. The promoter region encompassing 150 bp upstream of each haemolysis associated gene also was extracted, aligned and examined for SNPs uniquely present in whBh isolates.

The whole genome sequences were deposited in the European Nucleotide Archive under study Accession number PRJEB29439.

## Results

### Species confirmation and allocation to sequence types

Of the 39 newly sequenced isolates in this study, the four from SD cases, three from pigs with diarrhoea and one from a pig of unknown clinical background were strongly haemolytic on TSA containing 10% bovine blood, while the other 31 were all weakly haemolytic (Table 1). Molecular genetic examination by *nox*-RFLP [21], species-specific PCR [22, 41] and partial *nox*-gene sequencing using the same primers as for *nox*-RFLP-PCR was undertaken for species identification, and all 39 isolates were identified as *B. hyodysenteriae* by one or several of these tests (Additional file 1). Fourteen isolates (3 strongly haemolytic and 11 weakly haemolytic) also were tested by MALDI-ToF [24], and again all were identified as *B. hyodysenteriae* (Additional file 1). The 39 isolates were subjected to whole genome sequencing, and all were identified as *B. hyodysenteriae* using Kraken *k*-mer analysis [34] (Additional file 1). Whole-genome sequencing of the 39 newly sequenced isolates on average reached a depth of coverage of 86 reads (32–219). The genome size was 3.0–3.7 Mb with a GC content of 28.1–33.6% and encompassing 2617–2858 putative protein coding sequences. For the 39 genomes, 77–96% of the bases could be aligned to the 3 000 694 bp of the reference genome *B. hyodysenteriae* WA1 (Additional file 2). These genome properties were similar to the reference strain WA1 [12], and other published *B. hyodysenteriae* genomes [13, 17, 32, 33]. Prior to their inclusion in this study, a subset of the weakly haemolytic isolates had been identified biochemically as *B. intermedia* using standard biochemical protocols [27, 42, 43] (Additional file 1).

The multilocus sequence type (ST) was determined for each isolate from the WGS (Table 1), and the isolates were placed into a global context by reference to the *B. hyodysenteriae* MLST scheme [39]. Five isolates (two strongly haemolytic and three weakly haemolytic) could not be assigned to an ST because one or two of the seven house-keeping genes used for the MLST scheme were not detectable either by PCR amplification or by examining the genome sequences (Table 1). The remaining 34 isolates belonged to nine different STs, with seven strongly haemolytic isolates belonging to three STs. One weakly haemolytic isolate (JR88) was assigned to ST246, a new variant not previously represented in the MLST database, and the weakly haemolytic isolate D28 was assigned to ST172, as reported previously [18]. The remaining whBh isolates belonged to seven STs (Table 1). The STs of the weakly haemolytic isolates as identified by WGS corresponded to their STs in the PUBMLST database that had previously been identified by PCR amplification of the loci and sequencing. None of the STs containing the newly sequenced whBh isolates have previously been described outside of Europe.

### Genome analysis identifies a sub-clade of weakly haemolytic isolates

**Figure 1:**
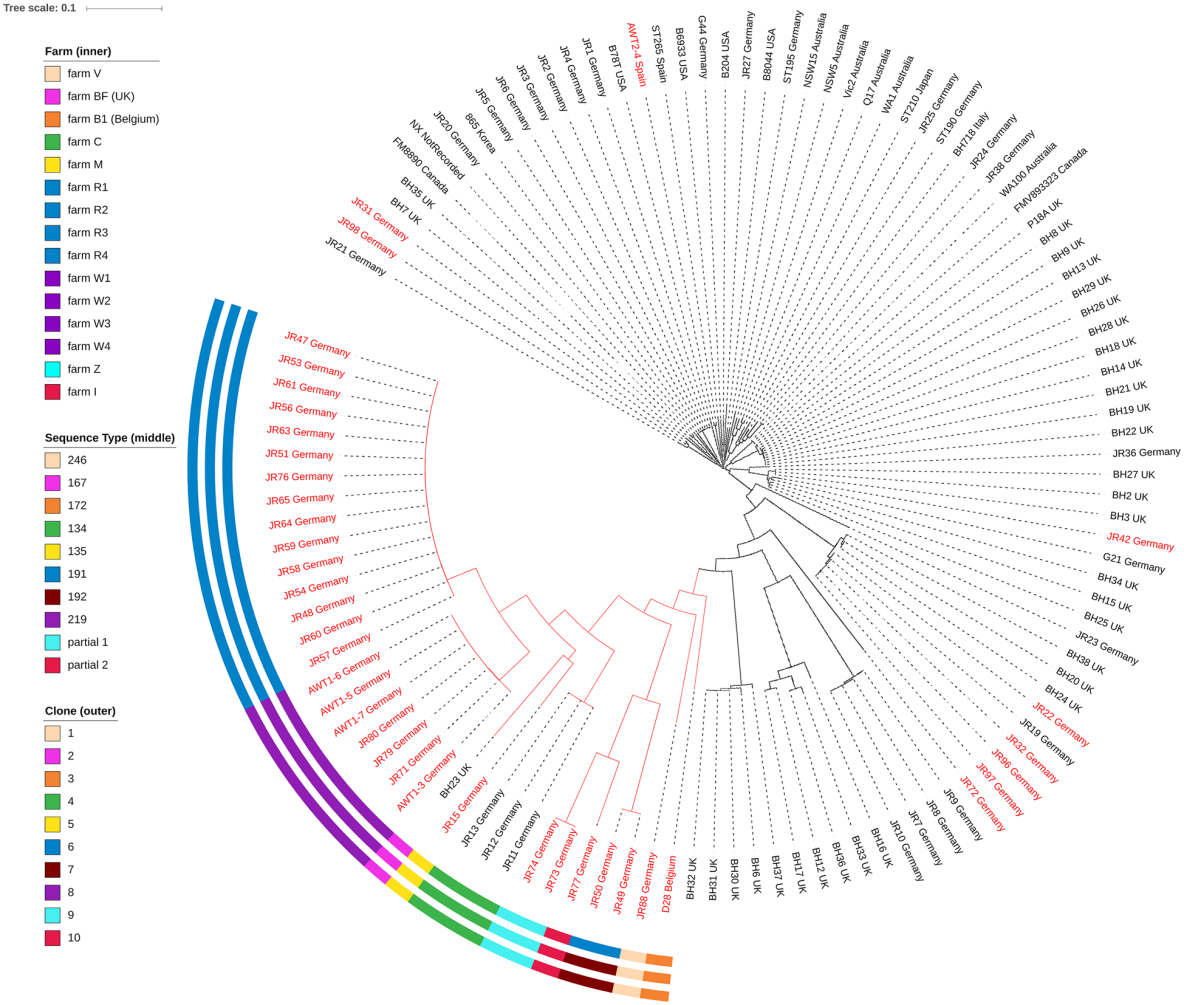
**A phylogenetic construction of** ***B. hyodysenteriae*** **isolates using a maximum-likelihood tree.** The 39 field isolates sequenced in this project are indicated with red font and have been included together with 77 published *B. hyodysenteriae* genomes. The sub-clade of weakly haemolytic isolates is indicated by red branches and for each weakly haemolytic isolate, the farm of origin (inner circle), sequence type (middle circle), and clone (outer circle) are presented in a colour-coded manner, as indicated in the figure legend and summarised in Table 1. The respective tree node of the sub-clade of weakly haemolytic isolates was supported with a bootstrap value of 100%. Image generated using the interactive Tree Of Life (iTOL) [53].

The phylogenetic relationships of the isolates were assessed using a maximum likelihood phylogenetic tree constructed using core genome SNPs from the WGS of the 39 new genomes together with 77 published *B. hyodysenteriae* genomes, including the genomes of four previously described weakly haemolytic isolates BH23, JR11, JR12 and JR13 [13, 17, 32, 33] (Figure 1). The seven strongly haemolytic isolates from Germany were most closely related to previously described German strongly haemolytic isolates: JR22, JR32, JR96, and JR97 clustered with published isolate JR19; JR31 and JR98 clustered with JR21; and JR42 clustered with G21 (Figure 1). Similarly, the strongly haemolytic Spanish isolate AWT2–4 grouped with the published strongly haemolytic Spanish strain ST265 (Figure 1). All but one of the 35 isolates that were weakly haemolytic on TSA formed a distinct sub-clade that also included the four previously sequenced whBh isolates [17, 32]. The respective tree node of this sub-clade of weakly haemolytic isolates was supported with a bootstrap value of 100%. Isolate JR72, which was weakly haemolytic on TSA, did not fall into this sub-clade and clustered separately from all other whBh isolates, which again was supported by a bootstrap value of 100% (Figure 1).

The phylogenetic tree further showed that within the weakly haemolytic sub-clade there were ten sub-clusters of whBh isolates, each comprised of a different ST (Table 1; Figure 1). Given the high sequence similarity in the core genome for members of a sub-cluster, each has been called a “clone”. Members of the clones had known epidemiological linkages and could be associated with particular farms or a set of related farms. For example, farms W1 to W4 represented a single epidemiological group from which seven whBh isolates of ST219 with ≤ 139 SNPs difference in their core genome were obtained. Farms R1 to R4 represented a different epidemiologically linked group, and 15 ST191 isolates (having ≤ 165 SNPs difference between isolates) were obtained from them. The ST192 isolates JR49 and JR50, having 107 SNPs difference, represented another weakly haemolytic clone. These were also recovered from Farm R1, which therefore had two different weakly haemolytic clones present, with different STs and > 17 000 SNPs difference between them. Isolates JR73 and JR74 were clonal (164 SNPs difference) and both were obtained from farm Z, which had no known epidemiological links to other farms in this study. German whBh isolates JR11, JR12, and JR13 from farm C belonged to the previously published ST134 [17], and differed from each other by ≤ 10 SNPs. The other five clones (each with a different ST) were each represented by a single isolate and came from farms with no known epidemiological links.

### Haemolytic activity of culture supernatants compared to agar culture phenotype

**Figure 2:**
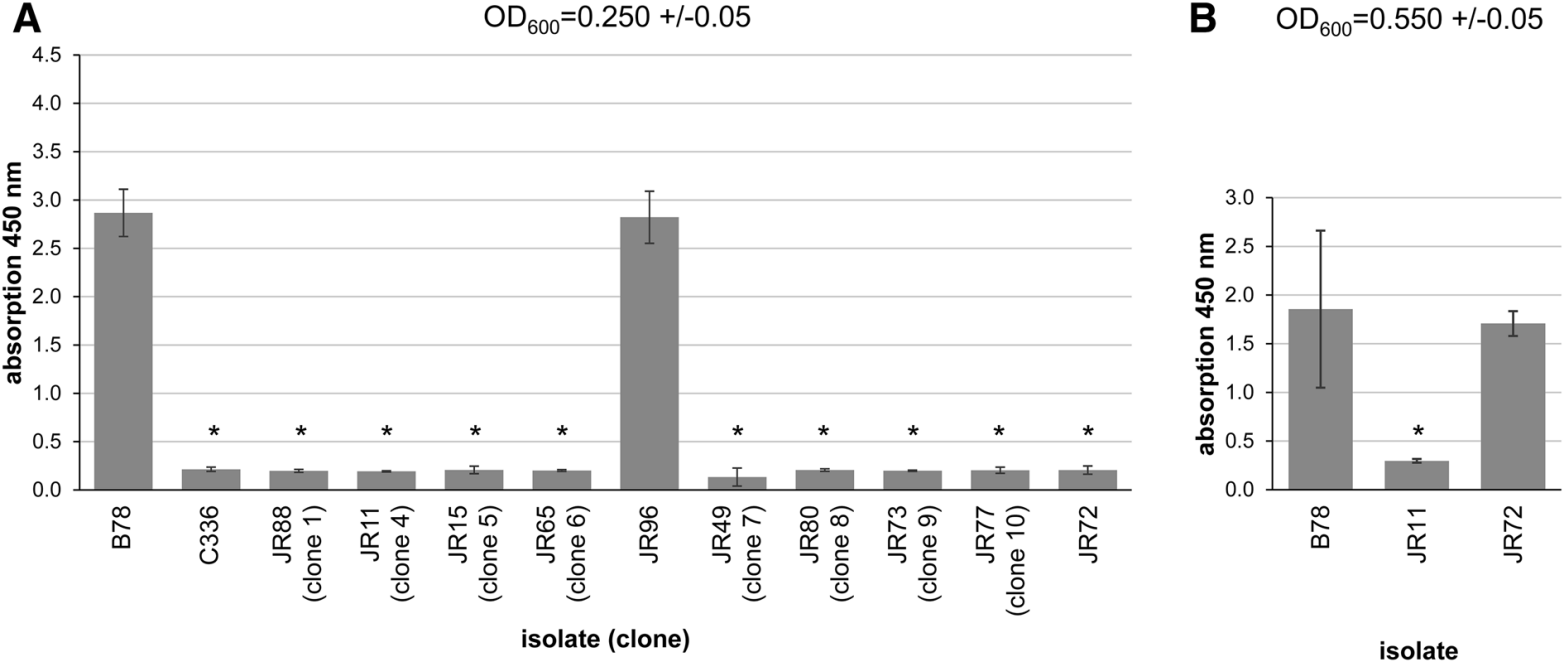
**In vitro haemolytic capacity of representative** ***B. hyodysenteriae*** **field strains.** Isolates were grown to an OD_600_ of 0.250 ± 0.05 (**A**) and field strains JR11 and JR72 and type strain B78^T^ were also grown to an OD_600_ of 0.550 ± 0.05 (**B**). Haemolysis is presented as the mean value of absorption at 450 nm after incubation of the red blood cell suspension with the supernatant of representative *B. hyodysenteriae* isolates. *B. hyodysenteriae* strain B78^T^ served as positive control and *B. innocens* strain C336 was the negative control. Isolates with a significant difference in haemolytic capacity compared to B78^T^ (*p* < 0.05 after Bonferroni correction) are indicated with an asterisk (*).

The weak haemolysis phenotype on TSA with 10% bovine blood of isolate JR72 and of one representative isolate from all clones except 2 and 3 was verified using an in vitro assay. All selected isolates, including JR72, had significantly decreased haemolysis compared to the strongly haemolytic *B. hyodysenteriae* type strain B78^T^ at OD_600_ = 0.250 ± 0.05 (Figure 2A). Isolate D28 (clone 3) has been tested previously in a similar system where it was compared to the strongly haemolytic *B. hyodysenteriae* strain B204, and it showed significantly decreased haemolytic activity in the same absorption range as the newly tested isolates [18]. Since JR72 was so different from the other weakly haemolytic isolates genetically, it and a representative of the other isolates (JR11) were tested in comparison to B78^T^ after growing to a higher optical density (OD_600_ = 0.550 ± 0.05). While JR11 still showed significantly decreased haemolytic activity, JR72 was as strongly haemolytic as B78^T^ (Figure 2B). Similar differences in haemolytic titres for *B. hyodysenteriae* isolates tested at different growth phases have been described previously [7]. Therefore, JR72 was classed as strongly haemolytic for the purposes of the comparative genome analysis. This analysis compared 34 weakly haemolytic isolates (30 from this study and the published genomes for BH23, JR11, JR12 and JR13 [17, 32]) with 82 strongly haemolytic isolates (9 from this study and 73 published genomes). All the latter were classed as strongly haemolytic even though this phenotypic trait was undocumented for some sequenced isolates that had been identified only using molecular methods.

### Weakly haemolytic *B. hyodysenteriae* possess mutations in haemolysin-associated genes and promoters

There are eight recognised haemolysin-associated genes in *B. hyodysenteriae*, which are considered to contribute to the typical strong haemolysis phenotype associated with this species [17, 18]. All eight genes were present in the 34 weakly haemolytic *B. hyodysenteriae* isolates examined. The predicted polypeptide sequences for each gene from both strongly and weakly haemolytic isolates were examined by alignment. This revealed a total of 41 different amino acid substitutions that were present only in weakly haemolytic isolates (and absent in all strongly haemolytic isolates), and hence possibly associated with the reduced haemolysis phenotype observed for these isolates (Additional file 3). Each weakly haemolytic isolate had amino acid substitutions in two to six haemolysin-associated genes, although no single substitution was present in every weakly haemolytic isolate. Eleven substitutions were common and present in at least 26 different isolates, whereas 11 other substitutions were each present only in one isolate. Although many of the amino acid substitutions were shared by several different weakly haemolytic clones, each clone had a unique combination of substitutions (Additional file 3). Most weakly haemolytic isolates possessed amino acid substitutions in haemolysin III (*n* = 32 of 34), haemolysin activation protein (*n* = 31), haemolysin III channel protein (*n* = 26), and haemolysin (*n* = 16). Few isolates had mutations in *hlyA* (*n* = 2), *tlyB* (*n* = 7), *tlyA* (*n* = 1), or *tlyC* (*n* = 1). Three isolates had amino acid substitutions in only two haemolysin-associated genes: JR15 (haemolysin III and haemolysin activation protein genes) and JR49 and JR50 (*tlyB* and haemolysin III). Only two isolates had a haemolysin III gene with no weakly-haemolytic specific amino acid substitutions (JR88 and JR77), but these had amino acid substitutions in four other haemolysin-associated genes. Analysis of haemolysin gene promoters revealed mutations unique to weakly haemolytic isolates in the promoters for haemolysin III (clone 7), haemolysin activation protein (clones 1, 2, 3, 4, 5, 6, and 8), and *tlyB* (clones 9 and 10), as summarised in Additional file 4. No SNPs unique to weakly haemolytic isolates were identified in the promoters of the five other haemolysin genes (*tlyA*, *hlyA*, hemolysin, *tlyC*, and hemolysin III channel protein).

### Absence of plasmid-borne virulence-associated genes is not unique to weakly haemolytic *B. hyodysenteriae*

The newly sequenced isolates were also examined for the presence of six genes present on the WA1 plasmid (locus IDs RS13000 to RS13025) which have previously been associated with virulence [44]. The isolates were placed into plasmid types based on the presence or absence of these genes, according to the scheme of La et al. [16]. As summarised in Table 1, four strongly haemolytic and three weakly haemolytic isolates were plasmid type 4, and consequently were missing two of these genes (locus IDs RS13000 and RS13005). All the remaining isolates, including four strongly haemolytic isolates, possessed none of these six plasmid genes and were thus assigned to plasmid type 1.

### Identification of genetic determinants associated with weakly haemolytic *B. hyodysenteriae*

A genome-wide association study (GWAS) was used to search for genes associated with either weakly or strongly haemolytic isolates. This identified two CDS significantly associated with strongly haemolytic isolates and five that were significantly associated with weakly haemolytic isolates (*p* < 2.7 × 10^−26^) (Additional file 5). The correspondence between these genes and the haemolysis phenotype was very strong: the two genes associated with strong haemolysis were present in all 81 strongly haemolytic isolates examined and absent in all weakly haemolytic isolates; similarly, the five genes associated with weak haemolysis were present in the 34 weakly haemolytic isolates and absent in the 81 strongly haemolytic isolates (Additional file 5). The genes associated with strong haemolysis had 100% nucleotide identity to the locus tags RS11510 (encoding a hypothetical protein) and RS11460 (encoding an ABC transporter permease) in the closed genome of *B. hyodysenteriae* strain WA1 [12], that was used as the reference sequence. Using blastn [37] the five genes associated with weak haemolysis were found to correspond to locus tags in the WA1 genome, but at lower nucleotide identities of 77–91% (Additional file 5). One CDS had 91% nucleotide identity to RS11460 and may represent an allelic variant of the RS11460 CDS identified in the strongly haemolytic isolates. An alignment of the predicted RS11460 amino acid sequence from all isolates showed that weakly haemolytic isolates had 92.1% amino acid identity compared to their strongly haemolytic counterparts and grouped separately on a phylogenetic tree (Additional file 6). Interestingly, the four other CDSs identified as unique to the whBh isolates were all located in close proximity to RS11460 on the assembled genomes, and corresponded to: RS11450 (hypothetical protein); RS11455 (ABC transporter ATP-binding protein); RS11465 (pseudogene); and RS13280 (pseudogene) (Additional file 5). The locus tags RS11465 and RS13280 are annotated as pseudogenes in the WA1 reference genomes, and they were not predicted as a CDS during Prokka annotation of assembled sequence data in all strongly haemolytic isolates. However, in all the whBh isolates they were predicted to be intact CDSs encoding a polypeptide, and therefore were not pseudogenes. The intact RS13280 CDS from weakly haemolytic isolates had 99% nucleotide identity to a CDS in *B. intermedia* strain PWS/A (Accession number CP002874; locus ID Bint_2317) predicted to encode a PadR-like transcriptional regulator. The intact RS11465 CDS from weakly haemolytic isolates had 96% nucleotide identity to a CDS in *B. intermedia* strain PWS/A (Accession number CP002874; locus ID Bint_2315) predicted to encode a hypothetical protein.

The core genomes were also screened by GWAS for SNPs uniquely present in whBh isolates. In total 69 SNPs were identified (*p* < 3.7 × 10^−25^), and, because the core genome had been prepared by mapping to the WA1 reference genome, each could be assigned a locus tag in WA1. This showed that 13 different CDSs carried SNPs associated with weak haemolysis (Additional file 7). Four of these CDSs were represented in the *B. hyodysenteriae* virulence gene list of Bellgard et al. [12]: RS02490 (TolC family protein; 10 SNPs); RS02985 (oligoendopeptidase F; 11 SNPs), RS09425 (peptidase T; 1 SNP); and RS11460 (ABC transporter permease; 16 SNPs). The hypothetical protein encoded by locus ID RS07825 had 22 SNPs and the eight remaining CDSs possessed ≤ 2 SNPs. Some of these SNPs may represent markers for the sub-clade, rather than having a role in the weakly haemolytic phenotype per se, and therefore require careful interpretation. The presence of multiple SNPs in a single CDS, a potential virulence function, and/or identification of the same CDS by gene-based GWAS would support a role for the CDS in contributing to the weakly haemolytic phenotype.

Four of the CDSs identified by SNP-based GWAS were predicted to encode ABC or TolC transporter functions, two encoded a peptidase, four encoded hypothetical proteins, and the remaining three encoded proteins with diverse functions, including one gene which encoded the DNA mismatch repair endonuclease MutL (Additional file 7). An alignment of the predicted TolC family protein encoded by RS02490 showed that the protein in the weakly haemolytic isolates had ~95 to 97.6% amino acid identity to RS02490 from strongly haemolytic isolates. The TolC family protein from the 34 weakly haemolytic isolates formed a distinct sub-group that was separate from the strongly haemolytic isolates (Additional file 8). Similarly, an alignment of the predicted MutL amino acid sequence from all isolates showed that weakly haemolytic isolates had from 89.5 to 96.9% amino acid identity to MutL in strongly haemolytic isolates. The MutL protein from 30 whBh isolates formed a distinct sub-group that was separate from the protein in the strongly haemolytic isolates (Additional file 9). The remaining four weakly haemolytic isolates possessed *mutL* genes that appeared to encode intermediate variants, which, together with JR72, grouped them between the other weakly and strongly haemolytic isolates (Additional file 9).

**Figure 3:**
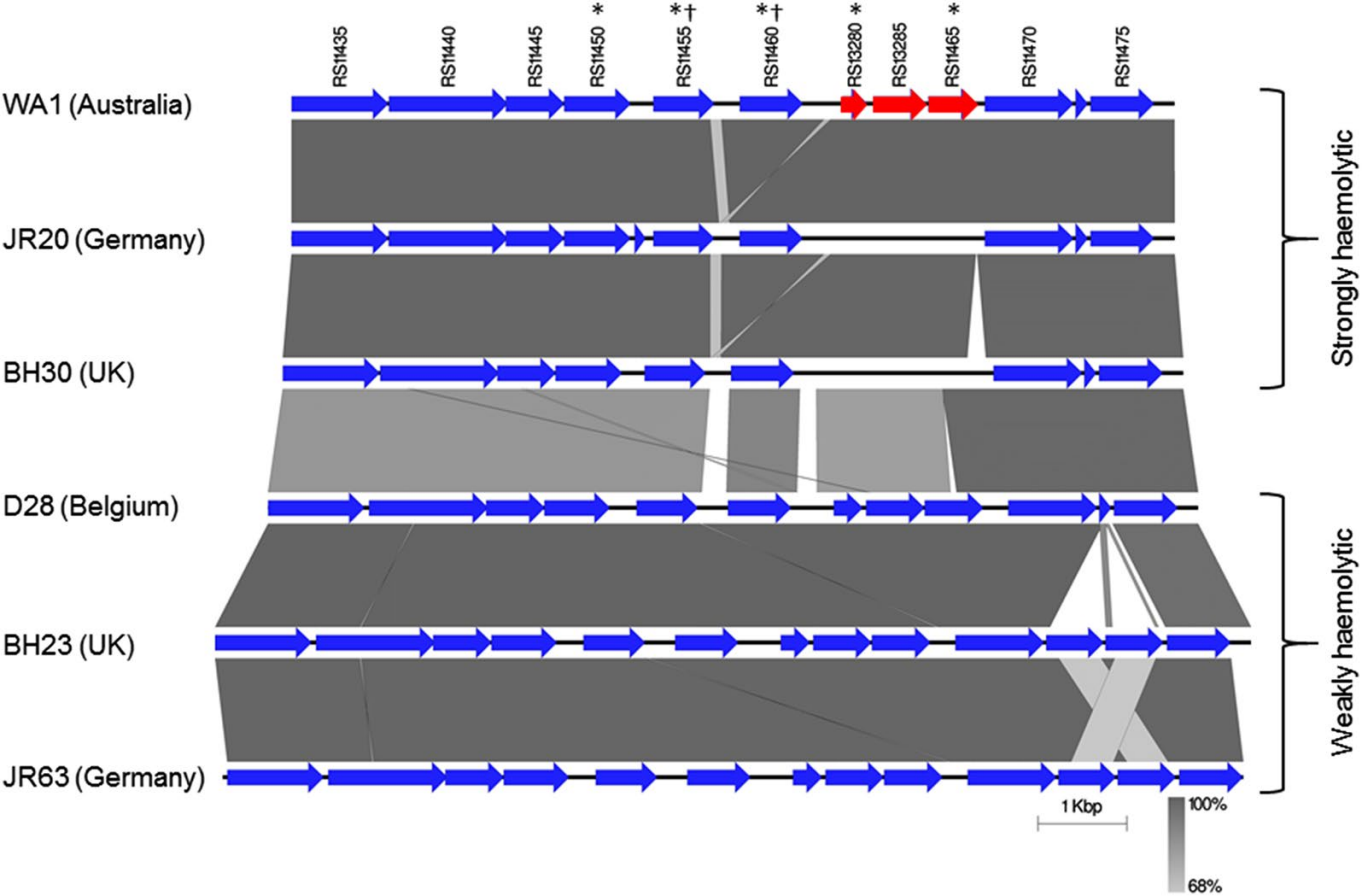
**Chromosomal arrangement of genes in** ***B. hyodysenteriae*** **showing differences between strongly and whBh.** *B. hyodysenteriae* genes are labelled according to the locus tag in the reference strain *B. hyodysenteriae* WA1 (Accession number NC_012225). Genes have been coloured to indicate intact genes in blue and pseudogenes in red. CDSs identified as significantly associated with haemolysis phenotype by gene-based and SNP-based genome-wide association studies are indicated with an asterisk (*) and dagger (†) respectively. Isolates WA1, JR20, and BH30 are presented as exemplar strongly haemolytic isolates. D28, BH23 and JR63 are presented as exemplar weakly haemolytic isolates. Regions of sequence similarity between isolates are shown by grey shading. Image generated using EasyFig [54].

It is noteworthy that the locus tags RS11455 and RS11460 were identified as having variants unique to weakly haemolytic isolates by both the gene-based and the SNP-based GWAS. Furthermore, three additional genes with differences between weakly haemolytic and strongly haemolytic isolates were located in the same region of the genome. The similarities and differences in gene synteny and nucleotide identity between weakly and strongly haemolytic isolates in this region are summarised for six exemplar isolates with different sequence types in Figure 3. This shows that strongly haemolytic isolates had three pseudogenes in this region, whereas in weakly haemolytic isolates these genes appeared to be intact. Nucleotide identity was lower between weakly and strongly haemolytic isolates than it was within weakly or strongly haemolytic groups.

## Discussion

*Brachyspira hyodysenteriae* is an important pig pathogen that causes significant economic loss, and consequently many pig producers have instituted rigorous programmes to detect and control it. Weakly haemolytic *B. hyodysenteriae* represent a new and emerging “atypical” variant of this pathogen, the presence of which can confound standard phenotypically-based identification methods and can cast doubt on the accuracy of routine molecular identification tests such as PCR. The genome analysis undertaken in this work verified that the studied weakly haemolytic isolates were indeed *B. hyodysenteriae*, confirming the results obtained by PCR and MALDI-ToF. Consequently, the initial biochemical identification of some isolates as *B. intermedia* was incorrect. This misidentification arose because *B. hyodysenteriae* and *B. intermedia* can share identical biochemical reactivity profiles and differ only by strength of haemolysis [23], highlighting the shortcomings of relying on biochemical and phenotypic identification alone for reliable identification of *Brachyspira* species.

An important finding of this study was that all 34 weakly haemolytic isolates belonged to a single sub-clade, consisting of 8 individual STs (three isolates could not be assigned an ST). A comparative genome approach was used to identify possible molecular genetic explanations for the difference in haemolysis. Examination of the eight recognised haemolysin-associated genes in *B. hyodysenteriae* that are believed to contribute to strong haemolysis, showed that these were present in all the whBH isolates, but that they all possessed non-synonymous mutations in two or more of these genes. Previous descriptions of amino acid substitutions in haemolysin-associated genes have compared weakly haemolytic isolates only to WA1 [17, 18]; however, in this analysis haemolysin-associated genes from 34 weakly haemolytic isolates were compared to the same genes from > 80 strongly haemolytic *B. hyodysenteriae*. This revealed that several amino acid changes previously identified as unique to weakly haemolytic isolates were actually present in one or more of the strongly haemolytic isolates in this larger panel. The changes identified in haemolysis-associated genes may account for the weakly haemolytic phenotype observed in culture and their reported putative lack of virulence in pigs, as has been proposed previously for whBh isolates [17, 18]. However, in the absence of structural data for these proteins it is not possible to predict the effect the identified amino acid changes may have on the activity of the proteins. Further work, possibly using mutant variants prepared in vitro, could be undertaken to obtain a more detailed understanding of the contribution that the identified mutations have on haemolytic activity in *B. hyodysenteriae*. However, *B. hyodysenteriae* is extremely recalcitrant to genetic manipulation and the authors are aware of only a very few publications describing gene replacement in this species [9–11, 45, 46]. This lack of methods impedes the scope to investigate gene function through generation of knock-out mutants or by replacement with variant alleles. Consequently WGS, together with robust epidemiological and phenotypic data, provides the most effective route to generate hypotheses on the genetic basis for phenotypes of interest for this species. Indeed, this approach has already been successfully applied to identify novel antibiotic resistance determinants in *B. hyodysenteriae*, including the new pleuromutilin resistance gene *tva*(A) [32].

Haemolysin proteins are exported across the bacterial cell wall and into the extracellular environment. In *E. coli*, export of haemolysin A (HlyA) is undertaken by a type I secretion pathway that involves an ABC transporter protein (HlyB), a membrane fusion protein (HlyD), and an outer membrane protein (TolC) [47]. The genes encoding HlyA, HlyB, and HlyD are in a linked single operon, while TolC is encoded elsewhere in the genome and is a component of several different export mechanisms, including the AcrAB-TolC efflux pump [48]. In *B. hyodysenteriae,* genes encoding the HlyB and HlyD proteins have not been defined, suggesting that a different export mechanism for HlyA, and possibly for the other haemolysin proteins, is present in this species. Our comparative genome approach identified a region of the *B. hyodysenteriae* genome containing five CDSs that consistently differed in nucleotide sequence between strongly and weakly haemolytic isolates (Figure 3). Two of these CDSs (RS11455 and RS11460) were predicted to encode ABC transporter proteins. Furthermore, an unlinked TolC family protein was identified as being consistently different in weakly haemolytic isolates. It is possible that these proteins have a role in the export of haemolysin(s) from *B. hyodysenteriae*. Additional investigations, such as gene expression and efflux pump activity assays, would be required to verify this hypothesis, but this was beyond the scope of the current study. Another important difference in this region of the genome was the presence of three CDSs in the whBh isolates that are pseudogenes in all strongly haemolytic isolates (and hence do not encode functional proteins). Although two of these intact CDSs from weakly haemolytic isolates encoded hypothetical proteins, one encoded a predicted PadR-like transcriptional repressor. PadR proteins are a large and diverse group of bacterial transcription factors that remain relatively poorly studied, although they have roles in the stress response in some Gram positive bacteria [49], in virulence gene expression in *Vibrio cholerae* [50], and in repression of efflux pump gene expression in the Gram positive species *Listeria monocytogenes* and *Lactococcus lactis* [51, 52]. It is therefore noteworthy that the PadR-like transcriptional repressor is present only in weakly haemolytic isolates, and its potential role for repression of haemolysis functions in *Brachyspira* species presents a new avenue of investigation.

Isolate JR72 provided an interesting exception, as it was strongly haemolytic at high cell density in the in vitro assays but weakly haemolytic at low cell density and on TSA. Similar differences in growth phase and haemolytic titre for *B. hyodysenteriae* isolates able to cause SD have been reported [7]. Although JR72 was obtained from a surveillance sample from a pig without clinical signs of SD, investigation of its potential to cause SD in experimentally infected pigs would be informative. JR72 was distantly related to the other whBh isolates (Figure 1), and may represent a separate lineage of *B. hyodysenteriae* with potentially compromised haemolysis capability. However, the underlying genetic mechanism(s) for its compromised haemolysis appears to be different to that of isolates in the weakly haemolytic sub-clade, as the GWAS analysis showed that JR72 possessed the variants seen in strongly haemolytic isolates. A separate GWAS analysis did identify two CDSs absent in JR72 but present in all other isolates, and 37 CDSs that were unique to JR72 (not shown). Furthermore, JR72 possessed five amino acid substitutions in haemolysin-associated genes that were present only in isolates from the weakly haemolytic sub-clade (not shown). With only a single isolate available it is difficult to confidently distinguish between markers for this lineage and traits associated with the compromised haemolysis phenotype.

Finally, it is interesting to speculate on the emergence of the weakly haemolytic *B. hyodysenteriae* sub-clade. The control of SD commonly employed for several decades entails measures such as antibiotic treatment, stringent cleaning and disinfection of premises, and on occasion depopulating the farm of all pigs following any identification of strongly haemolytic *Brachyspira*, especially in breeding or multiplier herds. A strong selective pressure has therefore been applied to *B. hyodysenteriae* which may have resulted in the evolution of variants with reduced haemolytic capability and/or virulence, which then persist in healthy pig populations not subjected to antibiotic treatments [16]. The presence of a mutated *mutL* gene in the weakly haemolytic isolates may indicate a reduced capacity for DNA repair and resultant increased genetic variation upon which this selective pressure was exerted. The emergence of the whBh sub-clade in Europe could have been favoured by individual members having reduced virulence, having an atypical phenotype (resulting in them being easily overlooked when focusing on strongly haemolytic spirochaetes in monitoring or diagnostic samples), and/or the establishment of the sub-clade in herds at the top of the breeding hierarchy. Such isolates may persist at the top of the breeding pyramid and may be then distributed downstream through the production chain. Two isolates of whBh also have been described in Australia [16], but these belonged to two different sequence types (ST151 and ST161), which are both genetically distinct from the European STs. In future, genome comparison of these Australian isolates would enable investigation of whether the weakly haemolytic phenotype has evolved independently on different continents, or if the European and Australian isolates potentially derived from the same weakly haemolytic ancestor.

As a result of the uncertain clinical significance of whBh, their presence in pigs causes considerable concern in the pig industry, impairs pig trade, and complicates pathogen surveillance by traditional methods. Weakly haemolytic isolates have not been reported in cases of SD to date, but their presence confounds interpretation of routine molecular surveillance data. Analysis by WGS was shown to be a powerful tool to characterise *B. hyodysenteriae* isolates, providing definitive species identification that verified previous molecular testing. The sub-clade of weakly haemolytic isolates were genetically diverse with respect to core genome SNP analysis and in their haemolysin-associated genes. However, all whBh isolates in the sub-clade possessed identical genetic variants in several genes that were not present in strongly haemolytic isolates, and which were likely to contribute to the weakly haemolytic phenotype. These genes provide useful targets for development of discriminatory molecular tests needed in SD surveillance. In the future, WGS can continue to be applied to provide detailed insight into *B. hyodysenteriae*, including establishing relationships between isolates, identifying factors associated with virulence and predicting antibiotic susceptibilities, as recently described [32]. In the absence of WGS, the combined use of *Brachyspira* culture and haemolysis testing, with molecular identification of weakly haemolytic as well as strongly haemolytic isolates, is recommended for comprehensive surveillance purposes, in herds with and without clinical SD.

## Additional files

**Additional file 1.** **Species identification results for the 39 isolates by various methods.**

**Additional file 2.** ***B. hyodysenteriae*** **strain sequence information, assembly statistics and gene content.**

**Additional file 3.** **Amino acid substitutions in haemolysis-associated genes present only in weakly haemolytic** ***B. hyodysenteriae*** **isolates.** Haemolysis-associated genes are identified by locus tag of the reference genome *B. hyodysenteriae* WA1 and name. Amino acid substitutions are presented using the amino acid single letter code: first letter is amino acid in *B. hyodysenteriae* WA1, number gives position and last letter is amino acid in weakly haemolytic isolate.

**Additional file 4.** **Summary of number of SNPs identified in the promoters of haemolysin-associated genes which are present only in weakly haemolytic isolates.**

**Additional file 5.** **CDSs identified by genome-wide association studies as unique to strongly or weakly haemolytic isolates.** The % cover and % identity were determined by blastn.

**Additional file 6.** **Phylogenetic tree for amino acid sequences of** ***B. hyodysenteriae*** **WA1 locus ID RS11460 encoding an ABC transporter permease.** The CDSs were extracted from the WGS of each isolate and aligned as amino acid sequence using ClustalV in MegAlign (DNASTAR). Weakly and strongly haemolytic isolates are indicated in the tree.

**Additional file 7.** **Summary of CDSs containing SNPs unique to weakly haemolytic isolates identified in the core genome by genome-wide association studies.** CDSs identified by locus ID in the *B. hyodysenteriae* WA1 reference genome.

**Additional file 8.** **Phylogenetic tree for amino acid sequences of** ***B. hyodysenteriae*** **WA1 locus ID RS02490 encoding a TolC family protein.** The CDSs were extracted from the WGS of each isolate and aligned as amino acid sequence using ClustalV in MegAlign (DNASTAR). Weakly and strongly haemolytic isolates are indicated in the tree.

**Additional file 9.** **Phylogenetic tree for amino acid sequences of** ***B. hyodysenteriae*** **WA1 locus ID RS00525 encoding the DNA repair protein MutL.** The CDSs were extracted from the WGS of each isolate and aligned as amino acid sequence using ClustalV in MegAlign (DNASTAR, Madison, WI, USA). Weakly and strongly haemolytic isolates indicated. The strongly haemolytic isolate JR72 is indicated in red font, and groups with the weakly haemolytic isolates.
