# Supplementary material for: Weakly haemolytic variants of Brachyspira hyodysenteriae newly emerged in Europe belong to a distinct subclade with unique genetic properties
Source: Vet Res. 2019 Mar 7;50:21. doi: 10.1186/s13567-019-0639-x (PMC6407217; doi:10.1186/s13567-019-0639-x)
Supplement: Supplementary file 6 — Additional file 6. Phylogenetic tree for amino acid sequences of B. hyodysenteriae WA1 locus ID RS11460 encoding an ABC transporter permease. The CDSs were extracted from the WGS of each isolate and aligned as amino acid sequence using ClustalV in MegAlign (DNASTAR). Weakly and strongly haemolytic isolates are indicated in the tree. [file 13567_2019_639_MOESM6_ESM.pptx]

## Slide 1
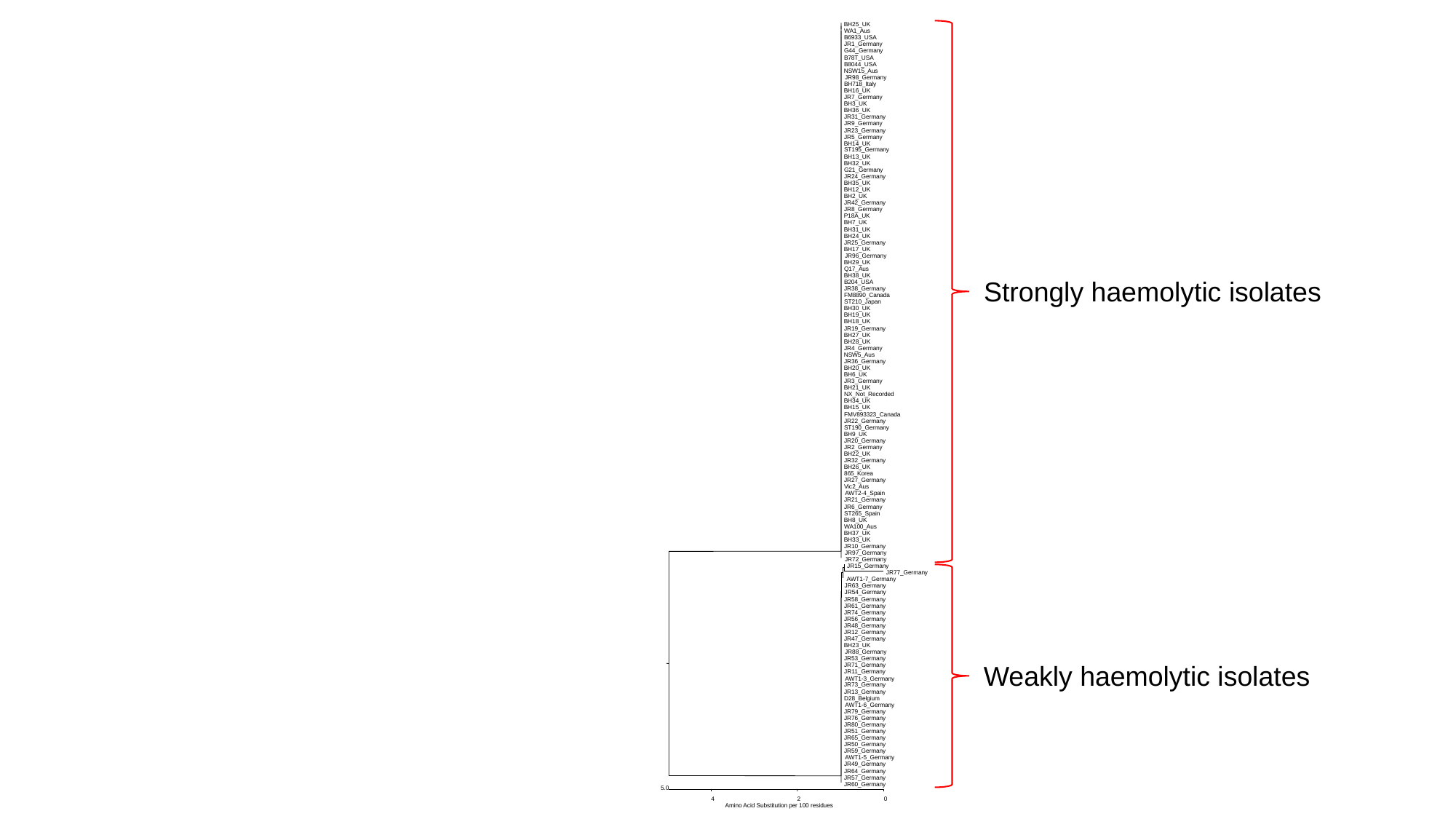

BH25_UK
WA1_Aus
B6933_USA
JR1_Germany
G44_Germany
B78T_USA
B8044_USA
NSW15_Aus
JR98_Germany
BH718_Italy
BH16_UK
JR7_Germany
BH3_UK
BH36_UK
JR31_Germany
JR9_Germany
JR23_Germany
JR5_Germany
BH14_UK
ST195_Germany
BH13_UK
BH32_UK
G21_Germany
JR24_Germany
BH35_UK
BH12_UK
BH2_UK
JR42_Germany
JR8_Germany
P18A_UK
BH7_UK
BH31_UK
BH24_UK
JR25_Germany
BH17_UK
JR96_Germany
BH29_UK
Q17_Aus
BH38_UK
B204_USA
JR38_Germany
FM8890_Canada
ST210_Japan
BH30_UK
BH19_UK
BH18_UK
JR19_Germany
BH27_UK
BH28_UK
JR4_Germany
NSW5_Aus
JR36_Germany
BH20_UK
BH6_UK
JR3_Germany
BH21_UK
NX_Not_Recorded
BH34_UK
BH15_UK
FMV893323_Canada
JR22_Germany
ST190_Germany
BH9_UK
JR20_Germany
JR2_Germany
BH22_UK
JR32_Germany
BH26_UK
865_Korea
JR27_Germany
Vic2_Aus
AWT2-4_Spain
JR21_Germany
JR6_Germany
ST265_Spain
BH8_UK
WA100_Aus
BH37_UK
BH33_UK
JR10_Germany
JR97_Germany
JR72_Germany
JR15_Germany
JR77_Germany
AWT1-7_Germany
JR63_Germany
JR54_Germany
JR58_Germany
JR61_Germany
JR74_Germany
JR56_Germany
JR48_Germany
JR12_Germany
JR47_Germany
BH23_UK
JR88_Germany
JR53_Germany
5.0
4
2
0
Amino Acid Substitution per 100 residues
JR71_Germany
JR11_Germany
AWT1-3_Germany
JR73_Germany
JR13_Germany
D28_Belgium
AWT1-6_Germany
JR79_Germany
JR76_Germany
JR80_Germany
JR51_Germany
JR65_Germany
JR50_Germany
JR59_Germany
AWT1-5_Germany
JR49_Germany
JR64_Germany
JR57_Germany
JR60_Germany
Strongly haemolytic isolates
Weakly haemolytic isolates
