# Supplementary material for: Weakly haemolytic variants of Brachyspira hyodysenteriae newly emerged in Europe belong to a distinct subclade with unique genetic properties
Source: Vet Res. 2019 Mar 7;50:21. doi: 10.1186/s13567-019-0639-x (PMC6407217; doi:10.1186/s13567-019-0639-x)
Supplement: Supplementary file 9 — Additional file 9. Phylogenetic tree for amino acid sequences of B. hyodysenteriae WA1 locus ID RS00525 encoding the DNA repair protein MutL. The CDSs were extracted from the WGS of each isolate and aligned as amino acid sequence using ClustalV in MegAlign (DNASTAR, Madison, WI, USA). Weakly and strongly haemolytic isolates indicated. The strongly haemolytic isolate JR72 is indicated in red font, and groups with the weakly haemolytic isolates. [file 13567_2019_639_MOESM9_ESM.pptx]

## Slide 1
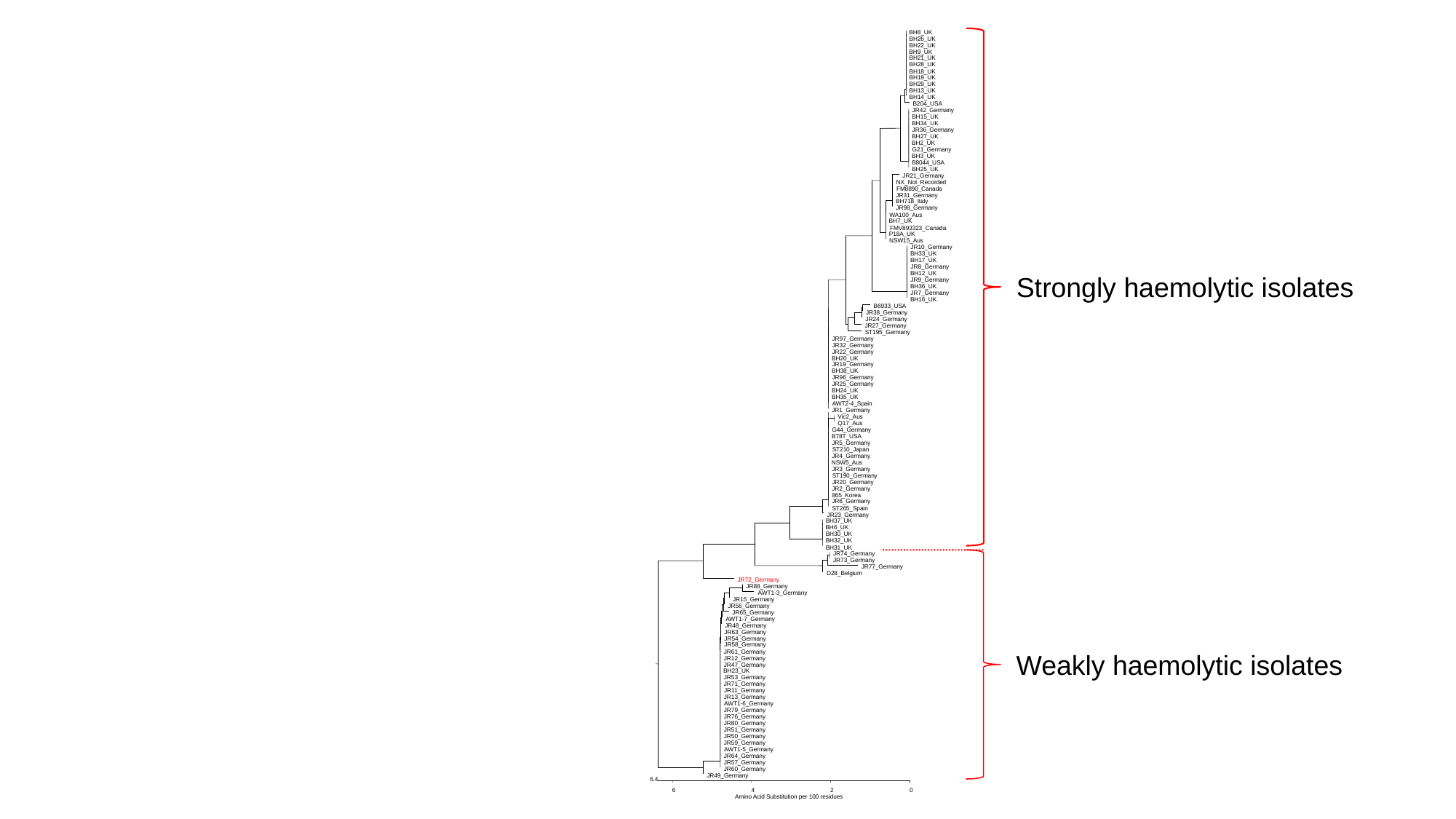

BH8_UK
BH26_UK
BH22_UK
BH9_UK
BH21_UK
BH28_UK
BH18_UK
BH19_UK
BH29_UK
BH13_UK
BH14_UK
B204_USA
JR42_Germany
BH15_UK
BH34_UK
JR36_Germany
BH27_UK
BH2_UK
G21_Germany
BH3_UK
B8044_USA
BH25_UK
JR21_Germany
NX_Not_Recorded
FM8890_Canada
JR31_Germany
BH718_Italy
JR98_Germany
WA100_Aus
BH7_UK
FMV893323_Canada
P18A_UK
NSW15_Aus
JR10_Germany
BH33_UK
BH17_UK
JR8_Germany
BH12_UK
JR9_Germany
BH36_UK
JR7_Germany
BH16_UK
B6933_USA
JR38_Germany
JR24_Germany
JR27_Germany
ST195_Germany
JR97_Germany
JR32_Germany
JR22_Germany
BH20_UK
JR19_Germany
BH38_UK
JR96_Germany
JR25_Germany
BH24_UK
BH35_UK
AWT2-4_Spain
JR1_Germany
Vic2_Aus
Q17_Aus
G44_Germany
B78T_USA
JR5_Germany
ST210_Japan
JR4_Germany
NSW5_Aus
JR3_Germany
ST190_Germany
JR20_Germany
JR2_Germany
865_Korea
JR6_Germany
ST265_Spain
JR23_Germany
BH37_UK
BH6_UK
BH30_UK
BH32_UK
BH31_UK
JR74_Germany
JR73_Germany
JR77_Germany
D28_Belgium
JR72_Germany
JR88_Germany
AWT1-3_Germany
JR15_Germany
JR56_Germany
JR65_Germany
AWT1-7_Germany
JR48_Germany
JR63_Germany
6.4
6
4
2
0
Amino Acid Substitution per 100 residues
JR54_Germany
JR58_Germany
JR61_Germany
JR12_Germany
JR47_Germany
BH23_UK
JR53_Germany
JR71_Germany
JR11_Germany
JR13_Germany
AWT1-6_Germany
JR79_Germany
JR76_Germany
JR80_Germany
JR51_Germany
JR50_Germany
JR59_Germany
AWT1-5_Germany
JR64_Germany
JR57_Germany
JR60_Germany
JR49_Germany
Strongly haemolytic isolates
Weakly haemolytic isolates
